# Quantitative and functional characteristics of monocytes and neutrophils in patients with treatment-resistant schizophrenia

**DOI:** 10.1192/j.eurpsy.2025.2039

**Published:** 2025-08-26

**Authors:** S. Zozulya, Z. Sarmanova, I. Otman, V. Kaleda, D. Tikhonov

**Affiliations:** 1 FSBSI “Mental Health Research Centre”, Moscow, Russian Federation

## Abstract

**Introduction:**

Despite significant progress in the treatment of schizophrenia, the number of patients with schizophrenia who do not respond to treatment remains constant. Identification of biomarkers of therapeutic resistance in the schizophrenia can help in early prediction of these conditions, as well as in the development of new approaches to treatment. Inflammation is considered as one of the possible mechanisms involved in the development of the pathological process in the formation of resistance to therapy in schizophrenia. The main cells of innate immunity, neutrophils and monocytes, are involved in the implementation of the inflammatory response.

**Objectives:**

To compare the subpopulation composition of monocytes and the level of other inflammatory markers in patients with treatment-resistant schizophrenia and in the control group.

**Methods:**

The study included 17 men with treatment-resistant schizophrenia (TRS) (27.0±8.0 years) and 15 healthy individuals without signs of mental and inflammatory diseases. The relative content of neutrophils and monocytes in the blood, as well as the ratio of monocyte subpopulations, estimated by the expression level of CD14 and CD16 receptors, were determined by flow cytofluorometry. The functional activity of neutrophils was determined spectrophotometrically by the activity of leukocyte elastase in plasma. The level of autoantibodies to S100B in plasma was estimated by ELISA.

**Results:**

A significant increase in the relative content of monocytes (U=28.0, p<0.01) and a decrease in neutrophils (U=35.0, p=0.036) were found in TRS patients compared to the controls. An increase in a proportion of the “transitional” CD14+CD16− subpopulation (U=61.5, p=0.04) and a decrease in the “classical” CD14++CD16− subpopulation (U=60.5, p=0.036) were accompanied by the proportion of “intermediate” inflammatory CD14++CD16+ and “non-classical” CD14+CD16+ subpopulations that did not differ from controls. A moderate increase in leukocyte elastase activity (U=34.0, p=0.001) and a high level of S100B autoantibodies (U = 55.0, p = 0.02) were found in blood plasma of patients. The proportion of “intermediate” CD14++CD16+ monocytes was negatively correlated with the level of autoantibodies to S100B (r= -0.55, p=0.021). It should be noted that this spectrum of immune parameters differs from the corresponding profile that we identified in patients with schizophrenia who responded to treatment. The main differences concern the proportion of “intermediate” monocytes, the relative content and functional activity of neutrophils.

**Conclusions:**

The identified quantitative and functional characteristics of monocytes and neutrophils in patients with TRS indicate the possible involvement of the cellular component of immunity in the development of resistance to treatment and may be associated with the severity of the disease in a long-term pathological process in the brain.

**Disclosure of Interest:**

None Declared

